# Annual Address of the President of Jefferson County, Alabama, Medical Society

**Published:** 1888-02

**Authors:** John D. S. Davis

**Affiliations:** Birmingham, Ala.


### ANNUAL ADDRESS OF THE PRESIDENT OF JEFFERSON COUNTY MEDICAL SOCIETY, JANUARY, 1888.

BY JOHN D. S. DAVIS, M. D., OF BIRMINGHAM, ALA.

*Gentlemen*:

I have tried hard to find a new line for an address and save you the burden of a personal retrospect.

I am reminded that the first medical society organized in the county was formed in the year 1865. It was soon disbanded, but re-organized in 1869 under the old Constitution. The society did but little until the year 1873 when it was revived to active work. I recall the work of the past and contrast it with that of 1887 to see wherein and how far we have improved.

My remembrance is vivid, and I shall speak of events which many of you have witnessed, many of the contrasts being already a part of the history of A abama, and which cannot, therefore, in any way be regarded as ancient.*

The members of the society did faithful and honorable service in the relief of suffering humanity during the cholera epidemic of 1873. The records and transactions of the State Medical Association of Alabama show that you were in a state of true activity at that time.

In passing this period in the history of the society, let us pause to reflect on the labors of our Sears, Luckie, Jordan and the lamented Taylor. These men of unselfish motives, untiring energy and perseverance, to whom we owe more than all the rest, for their constant labor to ameliorate the distressing condition of the men, women and children of this stricken city without reward, and for the sole purpose of bettering the condition of the unfortunate sufferers around them.

* History of Jefferson County, 1887, chapter v.—The Medical Profession, by Jno. D. S. Davis, M. D., pp. 97-119.

About this time, the county of Jefferson seemed to experience a "new birth." Just emerging from the dark gloom which so long hung heavily over her, shutting out from the world her treasures of iron and coal, at a time when the genius of invention and discovery gave intimation of her future triumph, when the nucleus was formed from which was to spring vitality and power sufficient to thrust her from the clouds of darkness into the sunshine of a resplendent noonday, she received the stroke of a blasting epidemic that prostrated the city of Birmingham to a barren waste.

With her people, who stood the severe ordeal and rallied to her rescue, were found three noble members of our profession performing their duty.

To their reward of conscious duty performed we add the praise of men.

*Rus in urbe.* Where but a few years ago the countryman plowed his furrow and hoed his row, we have erected to the memory of skill and energy this glorious, active, smoky, "magic" Birmingham. We have experienced a great change, and it is not restricted to the city alone, but quite as great a change in the profession of medicine, and there is yet room for much improvement.

A great bulk of work in medical organization, medical education, in surgery and gynecology, which for difficulty of detail, efficiency in medical examinations, and precision and brilliancy challenges the admiration of every sister society in the State, and is to our State organization the object of pride.

While this great center of iron wealth and the living activity of thousands of skilled laborers has so turned the scale of prosperity that the whole civilized world stands in awe and astonishment, I am reminded that the attention of the profession of the States has been attracted and is especially and eagerly waiting the results of united and consistent effort in Alabama in organizing and elevating the profession of medicine to the high standard of excellence we would achieve.

The State Medical Association of Alabama, with its organized county medical societies, is a prodigious organization for good, and its systematic, ritualistic and uniform regulations are carried out with the regularity and smoothness of clock-work. Like a well-oiled machine, the various county medical societies of the State, through their several boards of medical examiners, who by the duties imposed are stimulated to higher attainments and excellence, quicken and inspire the aspirant for a certificate to practice medicine in Alabama.

In a retrospect like this, it is an easy matter to speak of the wonderful advance made in the conception and development of such a State organization.

It was in 1873 that the State Medical Association of Alabama began its labors under a new charter, and soon became the legally authorized custodian of the sanitary regulations of this great commonwealth. Endowed with the power of authority, she stretched forth an eager hand to grasp the scepter of command.

Possessed of the ability to know the requirements of a newly-made doctor, and the power to exact evidence of such attainments,* the authority for obtaining such knowledge for Jefferson county, by written and oral examinations, was placed in your hands, and I congratulate you for your active co-operation and for the efficient and practical way your Board of Medical Examiners have conducted such examinations.

A careful examination of the records of your society and the transactions of the State Medical Association of Alabama will reveal convincing proof of the fact that your duties have been faithfully done.

Your members served in the battles that won over the legislators of Alabama and influenced them into the enactment of your medical laws. The victories which have attended your labors are due to pluck and pugnacity.

In the duties coming before your board of examiners, such high conception of the medical legislation of Alabama has been revealed as to attract the admiration of this whole society, draw forth the praise of the State Medical Association and the grate-

*See Medical Laws of Alabama.

ful thanks of an appreciative people. From the time your law went into effect, you have allowed no opportunity to pass unimproved to save the law becoming, as so often predicted, a dead letter. The high plane upon which your board placed a conception of the law won for you and them admiration, and for the law respect equaled in no other county of the State. By referring to your Transactions,* we learn that the high and lofty plane upon which your board of examiners was laboring, under the medical laws of Alabama, was lowered by the State Board of Medical Examiners to a level deserving the rebuke of a consistent people.

Your board recognized the object and intent of the law, and with no motive of selfishness or assured egotism pressed its claims for respect upon the profession and the people until the law was recognized as a fixture, and the reputation of your board has gone out all over the country as one, though just, to be feared.

The best illustration of the faithful work which placed your board of examiners in the lead in this work in the State is to quote from the report of the State Board of Censors of 1884, 1885 and 1886. In 1884 the examinations of the Jefferson county board were reported as the most rigid in the State. In 1885 the Jefferson county board was reported on as follows: "This board has insisted on a higher standard of qualifications than any other in the State." And again in 1886: "This board enjoys the enviable distinction of being the most efficient board in the State."

When the high esteem in which your board has been held has been considered, and, too, the requirements of the State Board in 1880,† do I not make just application in quoting the fitting axiom of Sydney Smith, who said: "It is not the man who first says a thing who deserves the credit, but he who says it so loud and so long that at last he persuades the world it is true."

There is an existing evil in our colleges, with a few exceptions, of admitting applicants to the study of medicine who are totally

---

* Alabama *Medical and Surgical Journal*, Vol. 1, No. 1, pages 268–279.

† Transactions of State Medical Association of Alabama, 1880. Also *Alabama Medical and Surgical Journal*, Vol. 1, p. 375.

ignorant even of a primary education. Omitting exceptions, the students of medicine of Alabama, for all time previous to 1874, were men of poor educational training. With the offices of our physicians and the medical school of our State filled with such material, is it little wonder that the profession so long remained below par? Dr .Edward Geddings, of the Medical College of Georgia, during his last lecture, delivered before the graduating class of 1878, recognizing, no doubt, the poor educational training of the students before him, remarked, "God pity the people of Georgia, for we are going to turn a band of wolves loose among them." The logical inference was that the graduating class would be worthy of little confidence and less respect.

In Germany our medical training is not recognized. The Germans actually have a supreme contempt for American medical schools and medical teaching. And why is this? Many of our American physicians go to Europe to finish their medical studies, who have had but poor literary training, and though graduates of American medical colleges, they have not education sufficient to gain the respect of educated German physicians, and hence the indignation of the Germans against American medical teaching.

"There is a class of American physicians equal to any in the world, but another, concerning whose general education the less said the better." It has been the determination of the State Medical Association of Alabama, under the existing law regulating the examination of applicants in some special branches of literature, who propose to begin the study of medicine, to get the co-operation of the various county medical societies and every physician of the State. Your board in this line of work has not been disposed to act in a dictatorial or autocratic manner; it recognized that its most legitimate sphere was in teaching applicants and persuading rather than even seeming to force them to avail themselves of its appliances for the better purpose of enabling them to prosecute more effectually their studies in medicine. The requirements of this law, if kept to the utmost extent by every physician in our State, by refusing to receive in their office for the study of medicine all who do not secure a certificate to begin the study of medicine in Alabama, can never materially effect the constant pouring into the profession the uneducated, unless the colleges all over this country are reformed to the necessity of, and the exactment of, some literary requirements before allowing a student to matriculate. It is a painful fact that the mass of the material out of which a large number of doctors are made in this country scarcely know more than how to read and write. It was during my service as member of the board of medical examiners for St. Clair county, in 1880, that an applicant for a certificate to begin the study of medicine came before me for examination who could not bound his native State. The man, realizing that he would be properly rejected, did not finish his examination, but went at once to Louisville, Ky., and entered one of the medical schools of that city, whose standard requirement for matriculation is, like many other schools, so low as to draw the line on "color" alone—*et id genus omne.*

That man, after graduating in medicine, returned to Alabama and began the practice of medicine in Talladega, then an unorganized county, without examination. He is to-day enjoying the privileges of the profession of Alabama.

Another man, who refused to study medicine in Alabama because of his inability to get a certificate to begin the study of medicine, graduated from a Georgia school, and was admitted to practice medicine in Cullman, Alabama. This man, whose ignorance is idiotic, I heard, during a discussion on typhoid fever, give vent to the following very remarkable bombasticy: "Mr. President, I am before the convention to give a discussion of short duration, and for elucidation, I take the side of possession. The gentlemen who preceded me have dwelt with long duration upon sandy foundations, and in an examination of their proclamations, I have found great deviations from their anticipations, and owing to my indisposition, I feel but little emulation." I blush to tell you that this man occupied for three years the important position of member of a county board of medical examiners. His duties were, in part, to examine on chemistry and materia medica. It is a fact that he did not know the difference between a mixture and a solution.

It has been said that "medical men and medical schools encourage men to enter upon the study of medicine before they have learned even to read and write." This is not true regarding the profession of Alabama. Our college and profession encourage under legislation a better requirement of education than any other State, I believe, to entitle an applicant to a certificate to begin the study of medicine. The man who now begins the study of medicine in Alabama has this assurance—though his medical training may prove so defective as to exclude him from the profession of Alabama, he will not, wherever he may go, appear as an ignoramus before educated men.

The profession of Alabama has accomplished much for which it should be congratulated. Through its organized influence good medical legislation has been created that not only benefits the people, but that vests power in the medical profession not surpassed by any other State in the Union. It is through this organization that the gathering of vital statistics has reached a point of reliability and truth; that quarantine is placed in the hands of the competent; that literary and medical education in Alabama has been raised to a higher standard, and ethics respected.

It is a wise philosophy which teaches that science no longer raises man above the common wants and necessities of his nature, but it serves to supply his wants. Learning does not make man perfect, but does render imperfect nature comfortable. In this connection the name of Bacon is suggested as one to whom we owe more than any other man, not on account of his discoveries or inventions, for he made neither, but because he gave the world a great truth—the "philosopher's key," which has unlocked the doors of nature and permitted multitudes to look upon her secrets and profit by the revelation.

A writer gives the following description of this "key:" "Examine carefully minute and particular facts for the establishment of general laws that we may thereby promote the material and physical welfare of men—that is, master nature by induction that human life may be preserved and made comfortable; that is, investigate with only the useful in view; that is, advance in knowledge that the conditions of men and women may be ameliorated." This has been the stimulant imbibed by the intellects of Alabama. The promoters of the grand organization of which we form a part had not for their object State organization for State legislation alone, or prosecuted the work of higher literary and medical education for intellectual culture alone, but that the profession might be elevated to that high standard of proficiency as to fit it better for the service of man. It should be the sole purpose and desire of every physician that not in the conscious possession of the reward so much as he may be induced to use knowledge for the purpose of bettering his happiness and that of those around him.

In the legislative history of Alabama there is one bright spot, one oasis in the surrounding desert, on which I delight to linger while I gather courage and hope for the future. I refer to the triumph which led to the enactment of her medical laws. Honor, yea, thrice honor, to those who aided in an efficient way and so materially effected the legislation of our medical laws. To their genius and benevolence the profession and the people owe a living gratitude. There are some changes, however, in the law regulating admission of members to the profession necessary* before the profession can reach that high standard of medical attainments so much desired. *Mutatis mutandis*, incompetents can no longer find admittance to our ranks.

Mirabeau has said that "nothing is impossible to the person who can will. Is that necessary? That shall be." If, then, the law regulating the admission of members to the profession be proven defective, I see no reason why this society has it not in its power to practically demonstrate, by unweary and faithful work, the significance of the only law of success: Is it necessary? That shall be!

You will all cordially agree with me that the main credit and honor of what has been accomplished belong peculiarly and pre-eminently to the distinguished Cochrane, who has been a devoted and untiring leader from the outset. His work speaks for itself and needs no other orator.

---

* *Alabama Medical and Surgical Journal*, Vol. 1, No. 3, pp. 193-194.

It has been said* that in this country the most important changes of government have been decided by her literature long before such changes had been considered by her legislative bodies. As a country can be judged by its general literature, so can any special department by its special literature. Hence, it becomes the medical profession to look well to the improvement of its literature.

While you have done much in medicine and surgery practically, you have not encouraged this part of your work. Your discussions, which for a long time in your history remained far in the rear, have been materially improved and elevated. Much improvement should yet be made.

The publication of your proceedings in monthly or quarterly reports, as suggested by Dr. W. E. B. Davis in a recent resolution, I believe, would prove especially beneficial and healthful in stimulating your members to report many cases of interest occurring in their practice and contribute in a practical way to your proceedings. Thus every physician would have an incentive to keep a closer record of cases and to study them more thoroughly. Thereby a superior knowledge of medicine and surgery would be acquired, and in addition the faculty of expressing in terse and laconic language his observations and conclusions.

Think how different the position your medical literature would occupy in a few years if your members, encouraged by the regular publication of your proceedings, would only follow these rules. It has been truthfully said that "it is to this free intercommunication of facts and views that medicine and surgery are indebted for their respected status." Take from the medical profession of the world its literature, and you have shorn it of all its strength and power.

Dr. Glisan's conception was correct when, writing under the title, Quack and Doctor, he wrote:

> **" Knowledge deep, as ocean's mighty bed,
> And broad as universal space o'er head
> Must be; else like a shallow, narrow stream,
> It drieth up when hot the sun doth beam.

* *Alabama Medical and Surgical Journal*, Vol. 1, pp. 57—61.

No truer advance can physic ever make
If theories for facts we mostly take;
Our knowledge must with practice always join,
For gold withoult alloy would make soft coin.
When deeply ploughed the scientific field
Doth many grains of wheat quite often yield,
Though when the surface one but slightly chafes,
The soil more chaff than golden grain vouchsafes.
Our minds shall, like our crystal mountain streams,
Be sparkling clear, reflecting golden beams
As dazzling bright, if faithful we but run,
As those of diamond, star or noonday sun."

Contrasting your medical discussions of the past year with previous years, I can but acknowledge that, without any imputation of self-laudation, it has been my high privilege to preside during a year when your discussions excelled your whole previous history.

We often hear the familiar expression that certain individuals failed in life for the want of an opportunity to rise. And equally true regarding medical societies. The several objects of this society are laudable, and honor is due you for what has been accomplished in medicine and surgery in a higher standard of medical ethics, and for what has been done toward medical literature; but your literature is much like the godly discussions around many of the home firesides, seldom found beyond the bounds of your own domicile. I believe it my duty to recognize this want of activity on the part of the society and include the suggestion made as a method of correction.

This is an age of specialism, and I wish here to allude to the growing necessity on the part of the general practitioner of studying medical specialties.

The world lingers no longer in the shadows of the "dark ages," when Providence furnished Columbus, Luther, Newton, Kent, Shakespeare, Harvey and Bacon to start the world in its career of material and physical glory and blessings, but at an "age" where, by unceasing and intelligent study, we can attain a knowledge so accurate and thorough as to make it a familiar pastime to gather the early morning flowers of the gigantic intellects of the time. Yet, however learned we may become, however vast may become our experience, we cannot form an accurate opinion of every phase of human suffering; it cannot be gained by constant and life-long study; attain a superior knowledge and familiarity of every specialty in surgery, gynecology, diseases of the kidneys, of children, of the nervous system, ear, eye, throat and chest, which would render us proficient and reliable in all these several departments of medicine, as each and every department is to-day, for itself, interesting and consuming the whole labor and time of the most efficient and progressive scholars and workers of the age. What a vast and inconceivable harvest attends their genius, knowledge and wisdom, adding a charm to every special branch of medicine. Ah, is it not worth the labors of a life-time to get abreast in the principles and details, practical and theoretical, of a single branch of medicine?

It is a good principle to learn when you can, how you can, where you can, from every source you can, and if this rule was invariably acted upon, it would bring about an infinite satisfaction to professional work. I do not want to urge by this that every doctor should be thoroughly grounded in and fully abreast in all the special branches of medicine to apply it in its general or broad sense; that would prove an impossibility and only encourage a superficial knowledge in all.

It is well to remember, however, that the duty of the physician covers the recognition of the causes of disease and wrong conditions generally, but does not necessarily include the details of correction in every case of eye, ear, nose, throat, lung or kidney trouble, any more than he should be a sanitary engineer or practical plumber to become a sanitarian.

I would not have you imagine that I consider the doctor competent and entitled to the distinction of sanitarian because, by walking into a bath-room, smelling at a wash-basin or a water-closet, that he has made an examination from which he may give an opinion that may involve the expenditure of much money to correct, any more than I would have you suspect me of the grossness to respect as authority the opinion of a doctor, however exact he might be in the classification of insanity or mental diseases and correct definition thereof, without long personal and practical experience with the insane, who would appear before a court to testify as to the insanity or non-insanity of a particular individual. For, in the words of no less authority than Dr. P. Bryce, "it is an extremely difficult matter to give a full and correct definition of insanity or morbid phenomena of mind, for the mind itself takes such a wide range and its faculties are so complex that it is well-nigh impossible to give such a definition as would include all who were insane and exclude all who are sane." In such evidence experience alone can be relied on.

It is getting to be a common thing for the family physician to be counseled in health matters more than as a man to give physic; and while his practice is fast passing into the hands of specialists, his advice is sought more than ever, if it is only to ask what specialist the sufferer shall consult, and the position is one requiring much judicial acumen to decide whether the invalid shall be sent to the gynecologist, neurologist or oculist.

I do not think because a physician makes an amputation or cauterizes a uterus that he is entitled to recognition as a surgeon or gynecologist. But I do insist that every physician has it in his power to become proficient in some branch of medicine. At the beginning of his professional career, if he will select some special branch of medicine, suited to his inclination and talent, by persistent study and practice to the end, he will grow in knowledge, skill and reputation. This can be accomplished in a great measure (not exclusively, however) without neglecting his study and attention to general medicine.

Every physician represents a circle to which he is an authority; by a certain number he is considered second to none, by others second to the medical almanac. In the one if he decrees death, they die; in the other, trust to luck. If he formulates a rule for the preservation of health and life, this circle will follow it implicitly and preach it to others. If an operation is advised they submit to it with every confidence of a favorable result; if a nostrum is prescribed they swallow it without question.

"The doctors prey to die or linger,
Just as the doctor may determine."

How necessary, then, that the physician should know thoroughly the reason why of any rule that should guide his professional procedure. I do believe in the encouragement of a growing necessity of specialism, and any physician who will approach this proposition with his mind unbiased will be convinced with me that the physician who takes his degree and begins at once the faithful study of some special branch of medicine, with a few cardinal principles to guide him, theoretically and practically prosecuting the work, will find himself growing proficient, learned and in public favor constantly.

Facts concerning the disregard of ethics have been hard-worked and worn threadbare in this country long ago. But I revive the scene to view two classes of violations. It is far from my mission to insist on the enforcement of any rules or code on the "straining at the gnat and swallowing the camel"—defend the homœopath or quack, but suggest that we be "reasonably free from sin" before casting a stone. We hear a perfect storm about the empiric or quack, who systematically and extensively make use of the advertising columns of the secular and religious press, and a loud howl about some unfortunate fellow who has been driven to contract-practice to keep the wolf from the door; yet seeing that their own wonderful operation is correctly reported—an operation with a guarantee, too, of a cure for *so much fee.* It requires keenness of sight and discriminating powers beyond my possession to mark the distinction between the legitimate and "quack" advertising, or the difference between the physician who promises a cure for so much fee and the doctor who works for many men under contract for a fixed salary.

What would you think of a man who would, after receiving one of those beautiful little badges so often seen on the coat lapel of the counsellors of our State Medical Association, if he represented to his patients that he had thus been decorated on account of his eminence? Such has been the case. Is he better than the public quack advertiser, empiric and humbug? What else can he be?

Let this society ever discountenance the man who goes about filling the ears of the people with his reports of wonderful operations and cures as the vilest of advertisers. A physician's record should be his advertisement, and that to be heralded through the profession only. It has been said that "in the Hippocratic oath can be found the foundation of a code of ethics too pure for our degenerate times," and while this should not be so, I am grieved to say I am so convinced.

"Thank God, we can for our society claim
No ignominious, but a noble aim,
Protective tariffs! No! nor patent rights
We seek; protection only from those blights,
The quacks, who, locust-like, infest the land
By thousands. They upon street-corners stand,
With smooth and oily tongues, or blatant cries,
Retail their salves, their poisons, and their lies;
These daring tricksters do no means forego
With cunning skill to thrive on human woe!
But quacks there are of many sorts and kinds,
As dudes we see of many grades and minds;
Yet some there are, although not quacks by name,
For purpose useful still they are the same,
Who play the dodge of always something new
In drugs or skill, and only known by few,
To please the fancy of the present age,
When novelty, not worth, is all the rage—
The good, if new, they say he must abide
And some, to gain applause, that men may stare
And say, 'Behold the doctor over there
A genius is,' ignore all business sense,
Which, after all, is only common sense;
Be doctor void of this, no art or trick
By him inspires my confidence when sick."

In retiring from the responsible office for which you chose me, I desire to tender my grateful thanks for the consideration given my labors, and assure you that it has been my constant endeavor to justify the confidence reposed in me. In my efforts to promote the prompt and regular transactions of the business, I had to almost entirely depend upon the co-operation of the members of the society, and it is a very grateful acknowledgment that I could always rely upon your active support when right, and on your kind indulgence even when wrong. I have had frequent occasion to invoke your indulgence, but I trust you have had no just cause to complain that it has been abused.

